# Recombinase polymerase amplification combined with lateral flow biosensor for rapid visual detection of *Clostridium perfringens* in chicken meat and milk

**DOI:** 10.3389/fvets.2024.1395188

**Published:** 2024-07-01

**Authors:** Rui Tian, Feng Xie, Yuqing Liu, Guangjin Liu, Qingxia Li, Jinxiu Wang, Hongjian Zhang, Lei Dai, Wei Zhang

**Affiliations:** ^1^The Sanya Institute of Nanjing Agricultural University, Yabulun Industrial Park, Yazhou Bay Science and Technology City, Sanya, China; ^2^College of Veterinary Medicine, Nanjing Agricultural University, Nanjing, China; ^3^Institute of Animal Science and Veterinary Medicine, Shandong Academy of Agricultural Sciences, Jinan, China; ^4^Hainan Animal Disease Prevention and Control Center, Haikou, China; ^5^College of Agriculture and Animal Husbandry, Qinghai University, Xining, China

**Keywords:** foodborne pathogen, *Clostridium perfringens*, on-site detection method, recombinase polymerase amplification, lateral flow biosensor

## Abstract

**Aims:**

*Clostridium perfringens* is one of the major anaerobic pathogen causing food poisoning and animal enteritis. With the rise of antibiotic resistance and the restrictions of the use of antibiotic growth promoting agents (AGPs) in farming, *Clostridium* enteritis and food contamination have become more common. It is time-consuming and labor-intensive to confirm the detection by standard culture methods, and it is necessary to develop on-site rapid detection tools. In this study, a combination of recombinase polymerase amplification (RPA) and lateral flow biosensor (LFB) was used to visually detect *C. perfringens* in chicken meat and milk.

**Methods and results:**

Two sets of primers were designed for the *plc* gene of *C. perfringens*, and the amplification efficiency and specificity of the primers. Selection of primers produces an amplified fragment on which the probe is designed. The probe was combined with the lateral flow biosensor (LFB). The reaction time and temperature of RPA-LFB assay were optimized, and the sensitivity of the assay was assessed. Several common foodborne pathogens were selected to test the specificity of the established method. Chicken and milk samples were artificially inoculated with different concentrations (1 × 10^2^ CFU/mL to 1 × 10^6^ CFU/mL) of *C. perfringens*, and the detection efficiency of RPA-LFB method and PCR method was compared. RPA-LFB can be completed in 20 min and the results can be read visually by the LFB test strips. The RPA-LFB has acceptable specificity and the lowest detection limit of 100 pg./μL for nucleic acid samples. It was able to stably detect *C. perfringens* contamination in chicken and milk at the lowest concentration of 1 × 10^4^ CFU/mL and 1 × 10^3^ CFU/mL, respectively.

**Conclusion:**

In conclusion, RPA-LFB is specific and sensitive. It is a rapid, simple and easy-to-visualize method for the detection of *C. perfringens* in food and is suitable for use in field testing work.

## Highlights

- A rapid detection method for *C. perfringens* was established by combining RPA with LFB for the first time.
- The RPA-LFB method can be performed at a constant temperature of 37°C in less than 20 min. The results can be determined by the naked eye and are suitable for on-site testing.
- RPA-LFB has specificity and sensitivity, and can detect *C. perfringens* contamination in chicken meat and milk.

## 1 Introduction

*Clostridium perfringens*, a Gram-positive anaerobic bacterium of the genus *Clostridium*, was first isolated by William H. Welch in 1891 (1). *C. perfringens* is an important anaerobic pathogen that causes food poisoning in humans and intestinal diseases in animals (2). It causes disease mainly by producing toxins. *C. perfringens* is classified into seven toxin types (A–G) based on differences in the production capacity of toxins *α* (*cpa*), *β* (*cpb*), *ε* (*etx*), *ι* (*itx*), *cpe*, and *NetB* (3). *C. perfringens* is highly adaptable to the environment due to its sporulation properties and is widely found in meat products, soil, water sources, feed and feces (4).

*Clostridium perfringens* food poisoning ranks as the second most common foodborne illness in most developed countries (5, 6). For example, there are approximately one million cases of this food poisoning each year in the United States, causing annual economic losses of $400 million (7, 8). Food is one of the main transmission routes of *C. perfringens* (9, 10), which mainly involves high-protein foods, such as raw meat and meat juice that have not been thoroughly cooked (11). *C. perfringens* has a high risk of transmission to humans via chicken meat (12, 13) and is more likely to be transmitted to consumers due to improper storage of milk and dairy products (14). Therefore, detection of *C. perfringens* in food samples is needed to ensure food safety for consumers.

Currently, diagnostic methods for *C. perfringens* include isolation of pathogen followed by confirmation with molecular techniques. Isolation of pathogen is the “gold standard” recommended by the World Health Organization, but its use in the field is limited by the 8–12 h incubation time and the need for isolation and purification of cultured strains (15). Therefore, the development of a simple, rapid and accurate method for the detection of *C. perfringens* is important for field testing and routine monitoring.

In recent years, many molecular diagnostic techniques have been developed for the rapid detection of pathogens, Such as loop-mediated isothermal amplification (LAMP) (16), multiple cross displacement amplification (MCDA) (17) and rolling circle amplification (RCA) (18). The advantages of simplicity and rapidity of these methods have opened up a new way for nucleic acid detection. Recombinase polymerase amplification (RPA) is a novel nucleic acid rapid isothermal amplification technology (19). This technique uses four core proteins (recombinase, DNA helicase, single-stranded binding protein, and DNA polymerase) to amplify target genes under isothermal conditions ranging from 25°C to 42°C. RPA has the advantages of high sensitivity and simplicity of operation. The assay can be completed in 15–20 min and requires only a water bath or thermostat. In recent years, RPA assay has been reported to detect a variety of pathogens. For example, applied to the detection of *Staphylococcus aureus* (20), *Streptococcus lactis* (21), *Acinetobacter baumannii* (22), *Mycobacterium tuberculosis* (23), etc.

Lateral flow assays have attracted attention for their rapidity, inexpensiveness, and ability to detect pathogens on-site (24). A number of LFB products suitable for on-site detection are already available on the market. The MERCK (Germany) Singlepath series, for example, has introduced LFB products for the detection of foodborne pathogens such as *Campylobacter*, *Salmonella* and *Escherichia coli* (Singlepath^®^ *Campylobacter* Rapid test-104143; Singlepath^®^ *Salmonella* Rapid test-104140; Singlepath^®^ *E. coli* O157 Rapid test-104141) BioControl (United States) has launched the LFB product VIP® Gold Listeria (60037-40) for the detection of *Listeria monocytogenes*.

In this study, recombinase polymerase amplification was combined with lateral flow biosensor to develop a rapid, simple and intuitive assay for the detection of *C. perfringens* in chicken and milk. While ensuring sensitivity and specificity, the method is more suitable for on-site detection in food production plants and food processing plants.

## 2 Materials and methods

### 2.1 Strains and cultures

*Clostridium perfringens* and food-borne bacterias were all stored in the WOAH Reference Laboratory of *Streptococcus suis*, Nanjing Agricultural University, China. The reference strains of *C. perfringens* types B, C, D, and E were gifted by Shandong Academy of Agricultural Sciences, China. The information of the strains is shown in Tables 1, 2. The reference strain of *C. perfringens* used for infection (ATCC13124) was resuscitated, cultured and purified according to Forti’s method (25). The frozen strain of *C. perfringens* was inoculated in cycloserine solid medium (TSC) (Haibo, Qingdao, China) and placed in an anaerobic environment (85% N2, 5% H2, 10% CO2). After incubation at 42°C for 12 h, the black rounded colonies were picked for purification and microscopic examination of Gram staining. *C. perfringens* microscopy was positive for Gram staining and showed straight and short rods. The purified colonies were cultured in FTG (Haibo, Qingdao, China) for liquid proliferation.

**Table 1:** Information on foodborne bacteria used in the study.

Strains	Species	Source	Separation date
ATCC13124	*Clostridium perfringens*	Reference strains	NA
ZWSA817	*Salmonella Gallinarum*	Chicken	2023.7.4
ZWST195	*Staphylococcus aureus*	Duck	2020.9.8
ZWEF03	*Enterococcus faecalis*	Bovine	2021.11.5
ZWEC1535	*Escherichia coli*	Chicken	2023.7.4
ZWVP249	*Vibrio parahaemolyticus*	Shrimp	2023.5.24
ZWYE026	*Yersinia enterocolitica*	Human	2022.4.3
ZWKP530	*Klebsiella pnenmoniae*	Human	2022.11.11
ZWRA232	*Riemerella anatipestifer*	Duck	2022.7.2
ZWVH062	*Vibrio harveyi*	Screw	2023.11.26

**Table 2:** Information on different toxin types of *C. perfringens.*

Strains	Species	Source	Type	Separation date
ATCC13124	*Clostridium perfringens*	Reference strains	A	NA
B6261	*Clostridium perfringens*	Reference strains	B	NA
C10720	*Clostridium perfringens*	Reference strains	C	NA
D8346	*Clostridium perfringens*	Reference strains	D	NA
E8084	*Clostridium perfringens*	Reference strains	E	NA
ZWCP160	*Clostridium perfringens*	Bovine	F	2022.11.5
ZWCP021	*Clostridium perfringens*	Chicken	G	2022.1.6

### 2.2 DNA extraction

The genomes of bacterial cultures were extracted according to the instructions of the TIANGEN Bacterial DNA Extraction Kit (DP302) (Beijing, China). The genomes of chicken and milk samples were extracted according to the instructions of the TIANGEN Processed Foods DNA Extraction Kit (DP326) (Beijing, China).

### 2.3 Design and synthesis of primers and probes

The *plc* gene (GenBank: L43548.1) of *C. perfringens* was used to design primers and probes using Primer Premier 5.^1^ When combined with lateral flow biosensor (LFB), the 5′ end of the reverse primer should be labeled with a biotin group, and the 5′ end of the probe should be modified with FAM. A dSpacer (tetrahydrofuran, THF) was labeled at the middle position of the 5′ and 3′ ends. The 3′ end is labeled with a blocking C3-Spacer modifying group. The primer and probe sequences are shown in Table 3 and were synthesized by ShengGong Bioengineering (Shanghai) Co., LTD.

**Table 3:** Primer and probe sequences for the RPA-LFB and PCR assay for detection of *C. perfringens.*

Primer	Sequence (5′-3′)
MIRA-F4	TCCATATCATCCTGCTAATGTTACTGCCGTTG
MIRA-R4	GTTACCTTTGCTGCATAATCCCAATCATCCC
MIRA-F5	TCAAGGGGTTTCAATCTTAGAAAATGATCTGT
MIRA-R5	GTTTATAGTTTCCTCTTTGCCATTCATATCTAGC
MIRA-R5-bio	[5′-biotin]-GTTTATAGTTTCCTCTTTGCCATTCATATCTAGC
Probe-5:	[5′-FAM]-AACTTAGAGATTTTAAAAGAGAACATGCAT-[THF]-GAGCTTCAATTAGGT-[3′C3spacer]
Plc-F	TGAAAAGAAAGATTTGTAAGG
Plc-R	AGTCTCAAACTTAACATGTCC

### 2.4 Screening of RPA primers and establishment of RPA-LFB assay

The genomic nucleic acid of *C. perfringens* reference strain ATCC13124 was used as the positive control, and the ultrapure water was used as the negative control. RPA amplification with two sets of primers was carried out at 37°C for 20 min. The amplified products were subjected to nucleic acid electrophoresis, and appropriate primers were selected for probe design.

A 50 μL reaction system was constructed, containing 25 μL of A buffer (including polymerase, recombinase, helicase, etc.) (TwistDx, UK), 2 μL forward primer and 2 μL reverse primer (10 μmol/L), 0.5 μL probe (10 μmol/L), 16 μL ultrapure water, and 2 μL DNA sample. After adding them to the reaction tube, 2.5 μL B buffer (MgSO_4_, UK, TwistDx) was finally added. After vortexing and mixing, the mixture was transiently centrifuged and incubated at 37°C for 15 min. After the reaction, the product was diluted 100 times with ultrapure water, and the product diluent was sucked by the lateral flow biosensor to read the results. The quality control line and the detection line were observed within 5 min, and the results were interpreted.

### 2.5 Optimization of RPA-LFB reaction conditions

The RPA reaction conditions were evaluated at different reaction times (10 min, 15 min, 20 min) and different temperatures (25°C, 30°C, 37°C, 42°C) to screen the best reaction conditions. The nucleic acid concentration of the positive sample in each reaction was 1 ug/μL. When reading the results, a blue line must be displayed on the quality control line of the LFB to confirm that the test was performed correctly.

### 2.6 Sensitivity tests and determination of detection limits

The positive nucleic acid samples were diluted into 7 concentrations (10 ng/μL, 1 ng/μL, 100 pg./μL, 10 pg./μL, 1 pg./μL, 100 fg/μL, 10 fg/μL), and ultrapure water was used as negative control for sensitivity test. Based on the sensitivity of the assay, the limit of detection (LOD) was first assumed to be the negative result multiplied by 10. The higher dilution (100 pg./μL) and lower dilution (10 pg./μL) were tested in parallel 20 times for each of the two concentrations. The concentration with at least 19 positive results is the limit of detection (LOD).

### 2.7 Specificity experiments and detection of *Clostridium perfringens* with different toxin types

*C. perfringens* and several other common foodborne pathogens (*Salmonella Gallinarum, Staphylococcus aureus, Enterococcus faecalis, Escherichia coli, Vibrio parahaemolyticus, Yersinia enterocolitica, Klebsiella pnenmoniae, Riemerella anatipestifer, Vibrio harveyi*) was used to evaluate the specificity of RPA-LFB. Ultrapure water was used as a negative control.

In order to investigate the ability of RPA-LFB to detect different toxin types of *C. perfringens*, we obtained seven toxin-types strains of *C. perfringens* (A, B, C, D, E, F, and G) with the help of the Shandong Academy of Agricultural Sciences, China. We used RPA-LFB to detect these different toxin types of *C. perfringens*.

### 2.8 Detection of *Clostridium perfringens* in chicken meat

In order to explore the detection ability of RPA-LFB for *C. perfringens* in chicken, we treated chicken meat according to Tian’s method (26). Raw chicken breasts purchased from supermarket freezers were aseptically cut into 1.5 cm × 1.5 cm × 0.5 cm squares (1.1 cm^3^). To sterilize chicken breasts, they were first immersed in 70% ethanol and sterilized for 15 min, the alcohol was eluted with sterile PBS, and then sterilized with UV light for 1 h. *C. perfringens* ATCC13124 was resuscitated and serially diluted (1 × 10^6^ CFU/mL, 1 × 10^5^ CFU/mL, 1 × 10^4^ CFU/mL, 1 × 10^3^ CFU/mL, 1 × 10^2^ CFU/mL). Fresh cultures of *C. perfringens* at various concentrations were centrifuged, washed and resuspended in ultrapure water. Chicken meat was artificially contaminated with ultrapure water resuspension of *C. perfringens* and incubated at 37°C for 1 h. Ultrapure water was used as the control group. Four samples were set for each concentration. DNA was extracted from each chicken meat sample and detected using RPA-LFB and PCR methods.

The reaction system for PCR was as follows: 12.5 μL of 2× Taq Mix premix (Novozymes Biotechnology Co., Nanjing, China), 1 μL each of forward and lower primers, 2 μL of DNA template, and 8.5 μL of ultrapure water. The sequences of primers (Plc-F, Plc-R) are shown in Table 3 and were synthesized by ShengGong Bioengineering Co., LTD (Shanghai, China). The reaction procedure was as follows: predenaturation at 94°C for 5 min, followed by 35 cycles of denaturation at 94°C for 30 s, annealing at 53°C for 30 s, and extension at 72°C for 30 s, followed by extension at 72°C for 10 min. The results of the amplification products were observed by 1.5% gel electrophoresis.

### 2.9 Detection of *Clostridium perfringens* in milk

The proportion of dairy cows infected with *Clostridium* is extremely high, and milk, as a carrier, is particularly important for food safety. In order to explore the ability of RPA-LFB to detect *C. perfringens* in milk, we treated milk with reference to Noor’s method (27). Pasteurized milk was purchased from the supermarket. *C. perfringens* ATCC13124 was resuscitated and cultured, and then serially diluted (1 × 10^6^ CFU/mL, 1 × 10^5^ CFU/mL, 1 × 10^4^ CFU/mL, 1 × 10^3^ CFU/mL, 1 × 10^2^ CFU/mL). Pasteurized milk (1 mL) was inoculated with freshly cultured *C. perfringens* at different concentrations (*C. perfringens* was treated as in Section 2.8), and ultrapure water was used as a control, and incubated at 37°C for 1 h. Four samples were set for each concentration. DNA was extracted from each milk sample and detected using RPA-LFB and PCR methods. Reaction conditions are the same as in Section 2.8.

## 3 Results

### 3.1 Results of RPA primer screening and establishment of RPA-LFB assay

**Figure 1:**
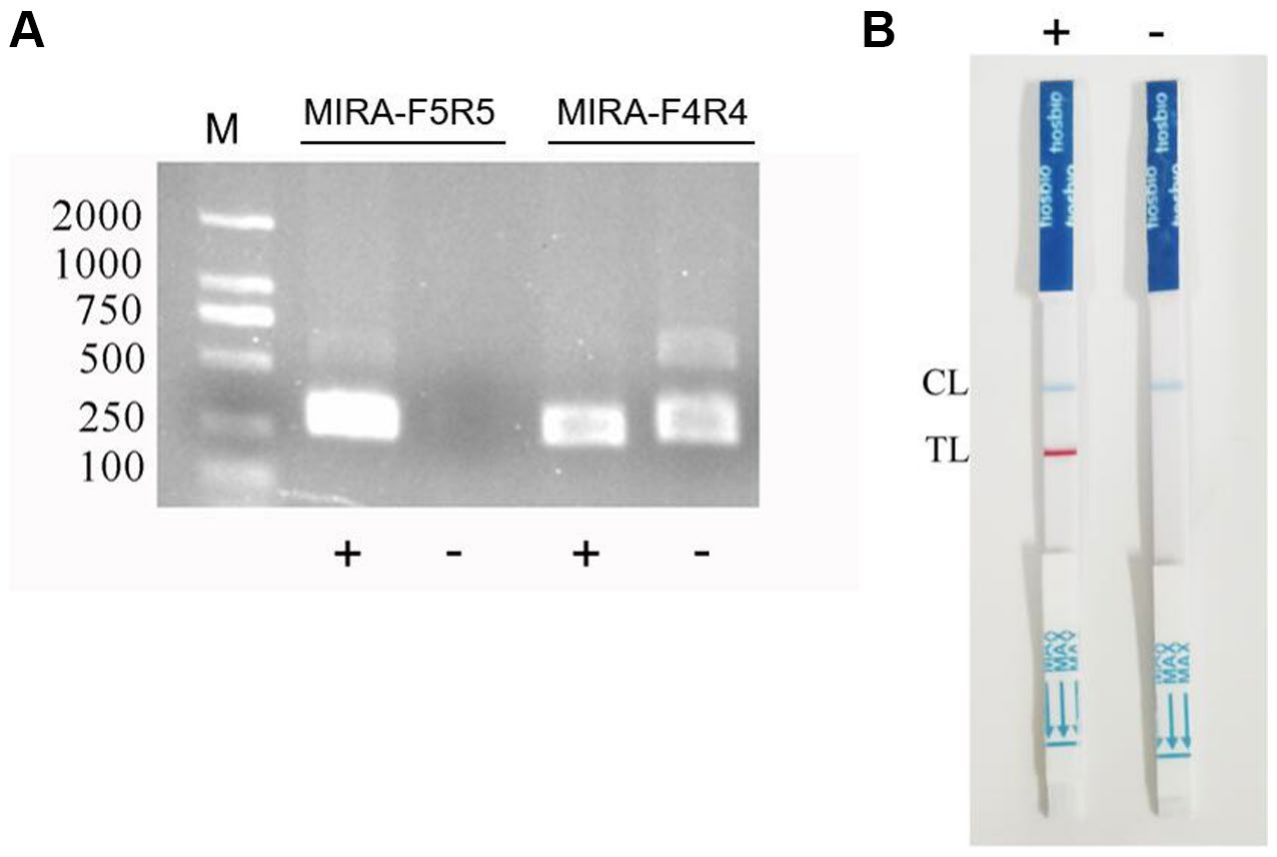
RPA primer screening and establishment of the RPA-LFB assay. **(A)** The RPA primers were screened by electrophoresis. **(B)** Negative and positive results of RPA-LFB.

The results of primer detection showed that the target band appeared in the positive samples of the two sets of primers, while no band appeared in the negative samples of primer MIRA-F5 and MIRA-R5, while the negative control products of primer MIRA-F4 and MIRA-R4 showed severe non-specific amplicons (Figure 1A). Therefore, primer MIRA-F5 and MIRA-R5 was selected for probe design.

**Figure 2:**
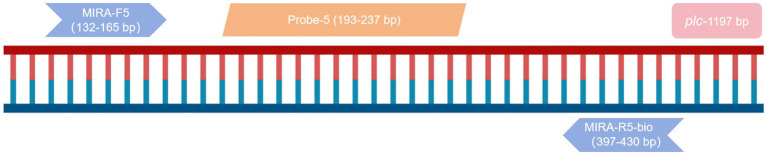
Schematic representation of RPA primers and probes aligned on the nucleic acid sequence.

The primer MIRA-R5 was combined with biotin labeling as a new reverse primer MIRA-R5-bio. The primers used in the RPA-LFD reaction were MIRA-F5 and MIRA-R5-bio. Probe-5 was used for the probe (Figure 2). The amplification was performed under the same reaction conditions for both the positive and the negative samples, and the amplified products were observed by LFB. The results showed that blue bands appeared in the quality control line of both samples, and red bands appeared in the detection line of the positive samples, while no bands appeared in the detection line of the negative samples, indicating that the RPA-LFB method was feasible (Figure 1B).

### 3.2 Results of optimization of reaction conditions

**Figure 3:**
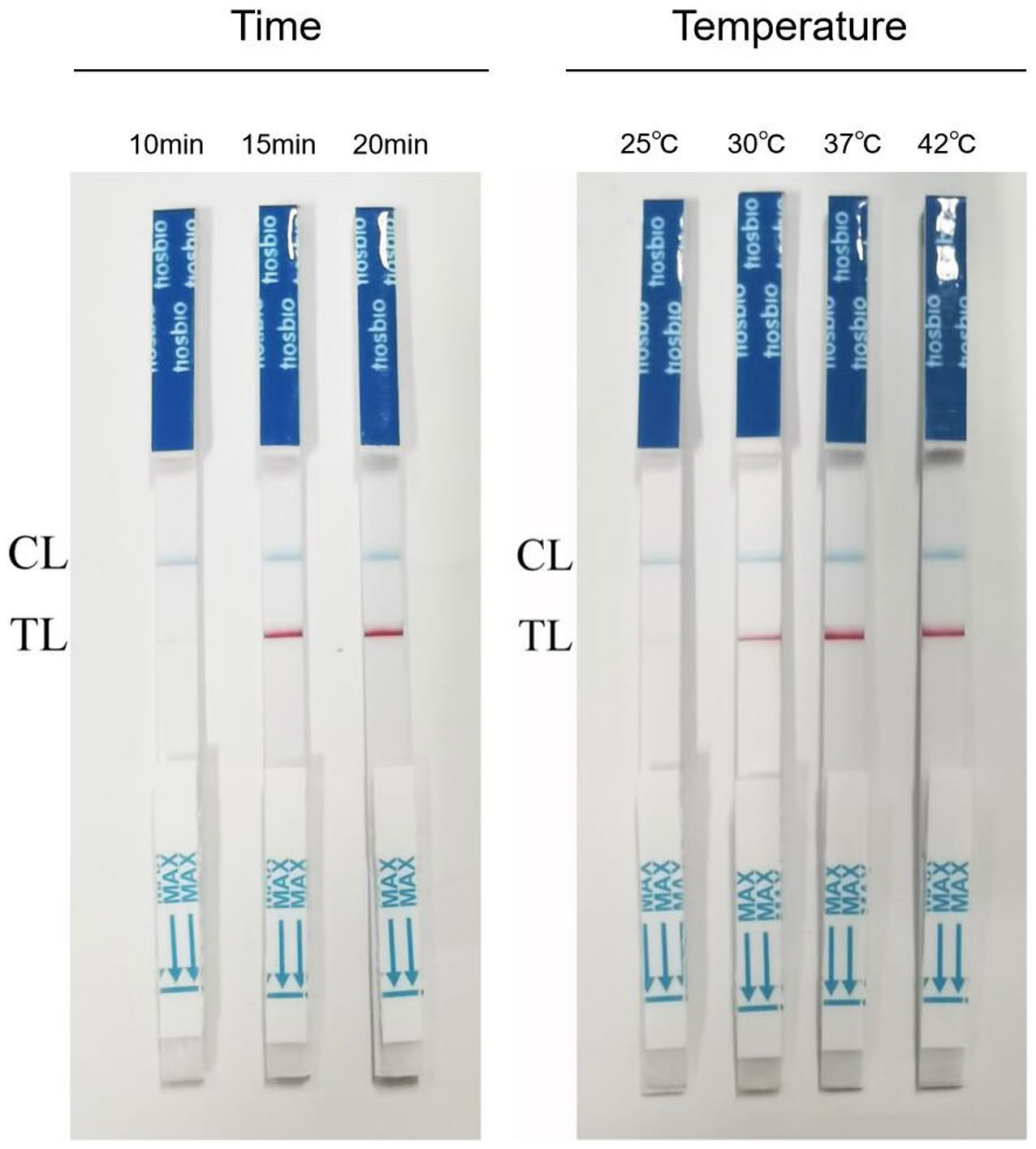
Results of RPA-LFB reaction time and temperature optimization.

Tests with different amplification times showed that RPA-LFB was able to react at 10 min, 15 min, and 20 min. However, when the reaction time was 10 min, the detection line only showed a weak red band. To ensure the stability of the results, we chose 15 min as the amplification time. The results of different reaction temperatures showed that RPA-LFB could be performed in the temperature range of 25–42°C. However, the color of the detection line was lighter at 25°C, and the color of the detection line was no longer deepened from 37°C. Considering the generality of the temperature conditions and the stability of the detection results, 37°C was selected as the reaction temperature (Figure 3).

### 3.3 Results of sensitivity and limit of detection

**Figure 4:**
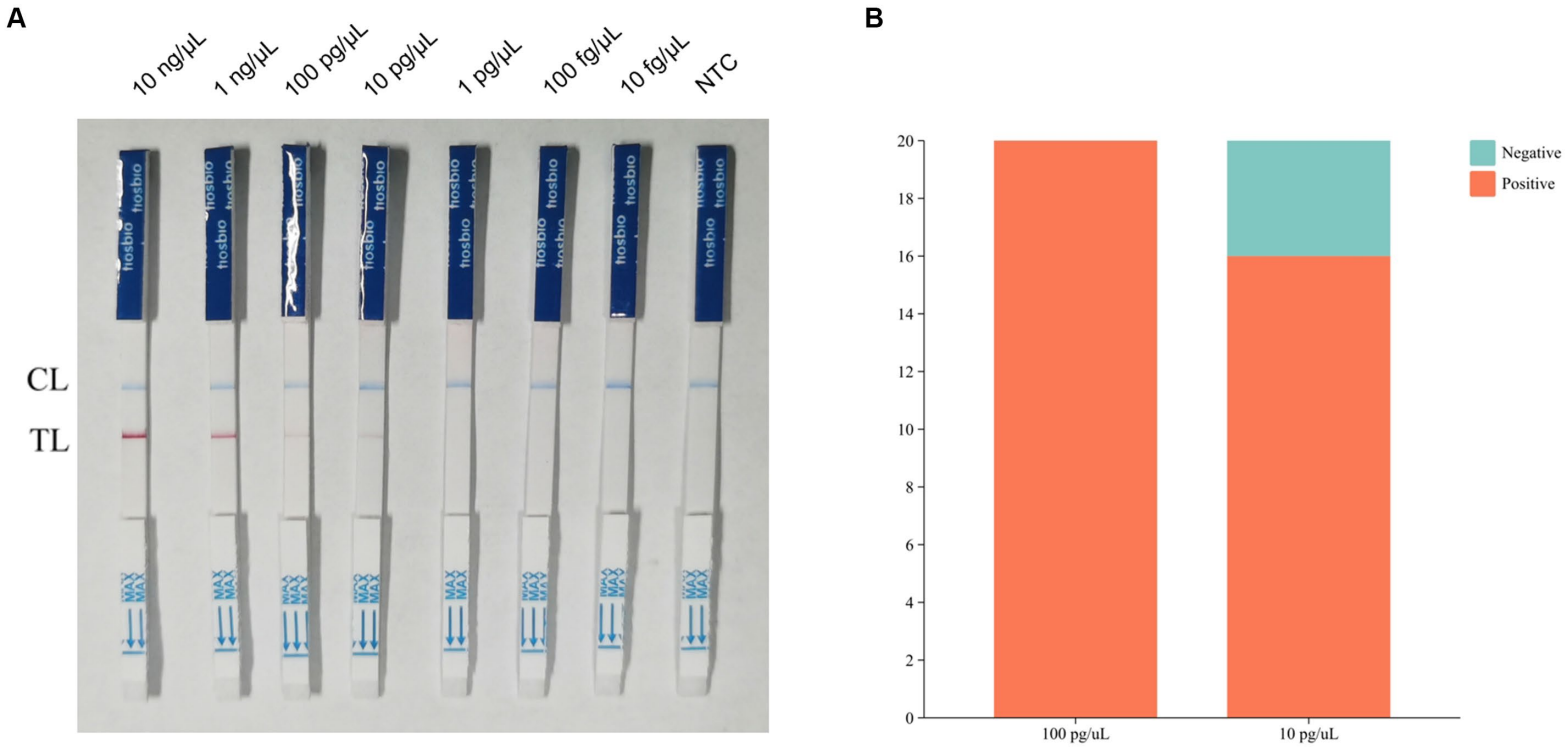
Results of RPA-LFB sensitivity tests and detection limits. **(A)** Results of RPA-LFB sensitivity testing. **(B)** Results of RPA-LFB detection limits.

The results of sensitivity showed that the assay was negative when the sample concentration was 1 pg./μL (Figure 4A). We assumed that the lowest detection limit of the RPA-LFB assay was 10 pg./μL. we chose concentrations of 100 pg./μL and 10 pg./μL for the determination of the detection limit. Twenty parallel assays were performed on nucleic acid samples at both concentrations using RPA-LFB. The results of the assay showed that all of the 20 samples with a concentration of 100 pg./μL had positive results. In contrast, four of the 20 samples with a concentration of 10 pg./μL had negative results, and the number of positive results was less than 19 (Figure 4B). Therefore, the detection limit of RPA-LFB was 100 pg./μL.

### 3.4 Results of specificity and detection of different toxin types of *Clostridium perfringens*

**Figure 5:**
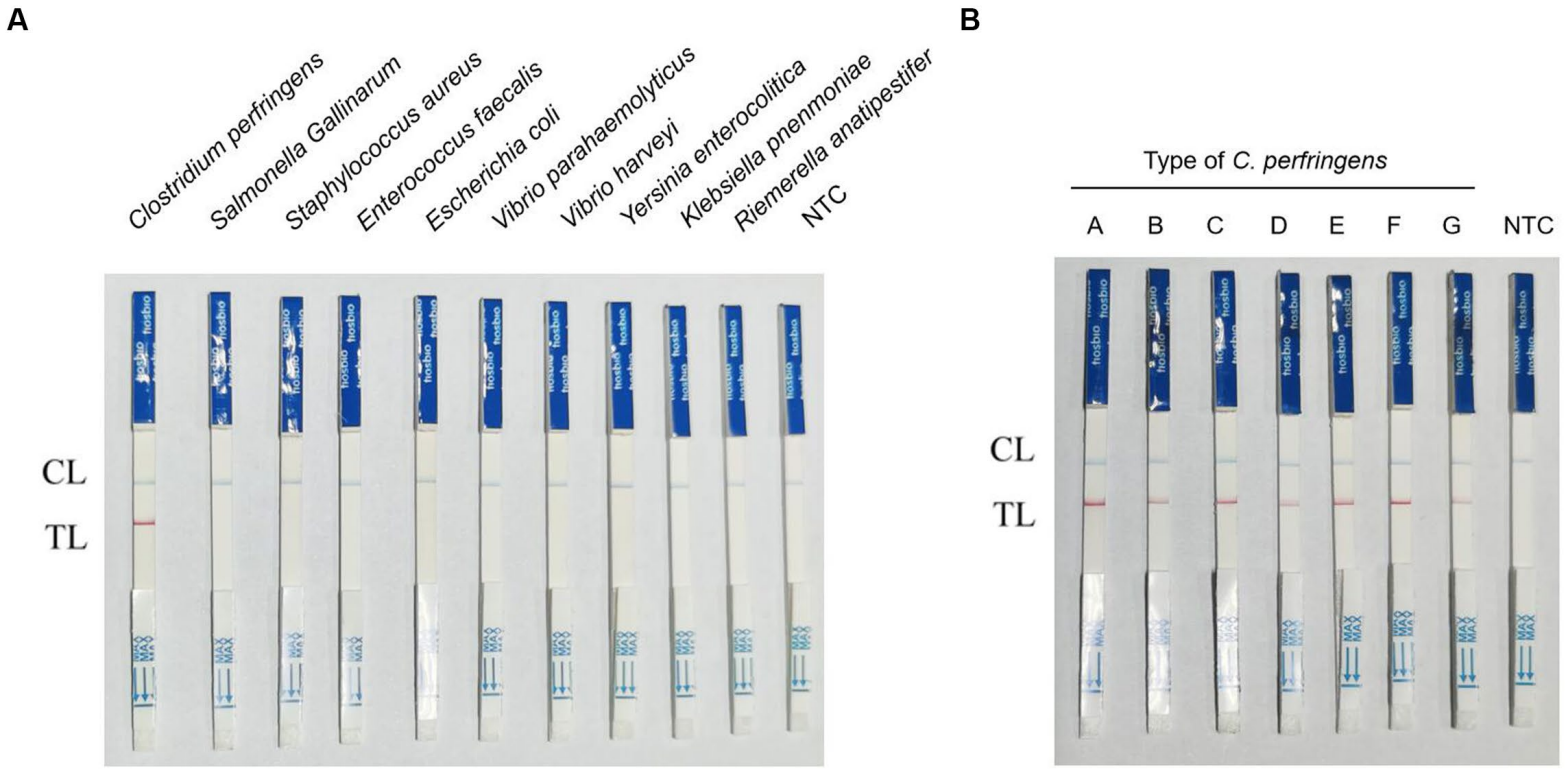
Results of RPA-LFB specific and detection of different toxin types of *C. perfringens*. **(A)** Results of RPA-LFB specific testing. **(B)** Results of RPA-LFB different toxin types of *C. perfringens.*

The results of the specificity test showed that of the 10 foodborne bacteria tested, only *C. perfringens* showed positive bands, indicating the specificity of RPA-LFB (Figure 5A). In the testing of seven different toxin types of *C. perfringens*, the samples of all toxin types showed positive because the *plc* gene was present and highly conserved in all toxin types of *C. perfringens* (Figure 5B). RPA-LFB can be used to detect various toxin types of *C. perfringens*.

### 3.5 Detection results of *Clostridium perfringens* in chicken meat

**Figure 6:**
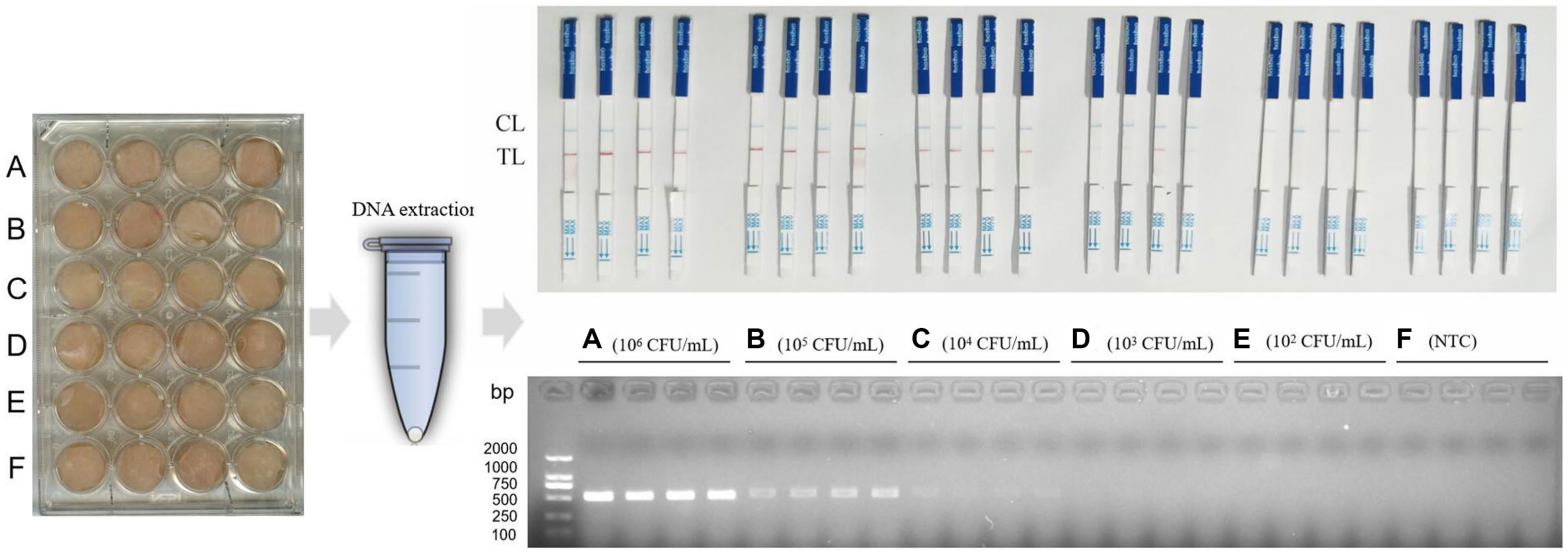
RPA-LFB detection of *C. perfringens* contamination in chicken meat.

In the chicken samples, when the concentration of artificially contaminated *C. perfringens* was 1 × 10^5^ CFU/mL, the results of RPA-LFB were positive, and the results of PCR electrophoretic bands began to show weak positivity. When the contamination concentration was 1 × 10^4^ CFU/mL, the PCR electrophoretic bands were no longer recognizable, while the RPA-LFD was still effective in detecting the bacteria. When the concentration of *C. perfringens* was 1 × 10^3^ CFU/mL, the RPA-LFB results were weakly positive, and the color of the red strip of the LFD was already very light at this time, the result was no longer stable at that time and might have an impact on the field test. When the contamination concentration decreased to 1 × 10^2^ CFU/mL, the results of both assays were negative (Figure 6). Therefore, we concluded that RPA-LFB was able to effectively detect *C. perfringens* contamination in chicken meat at a concentration of 1 × 10^4^ CFU/mL.

### 3.6 Detection results of *Clostridium perfringens* in milk

**Figure 7:**
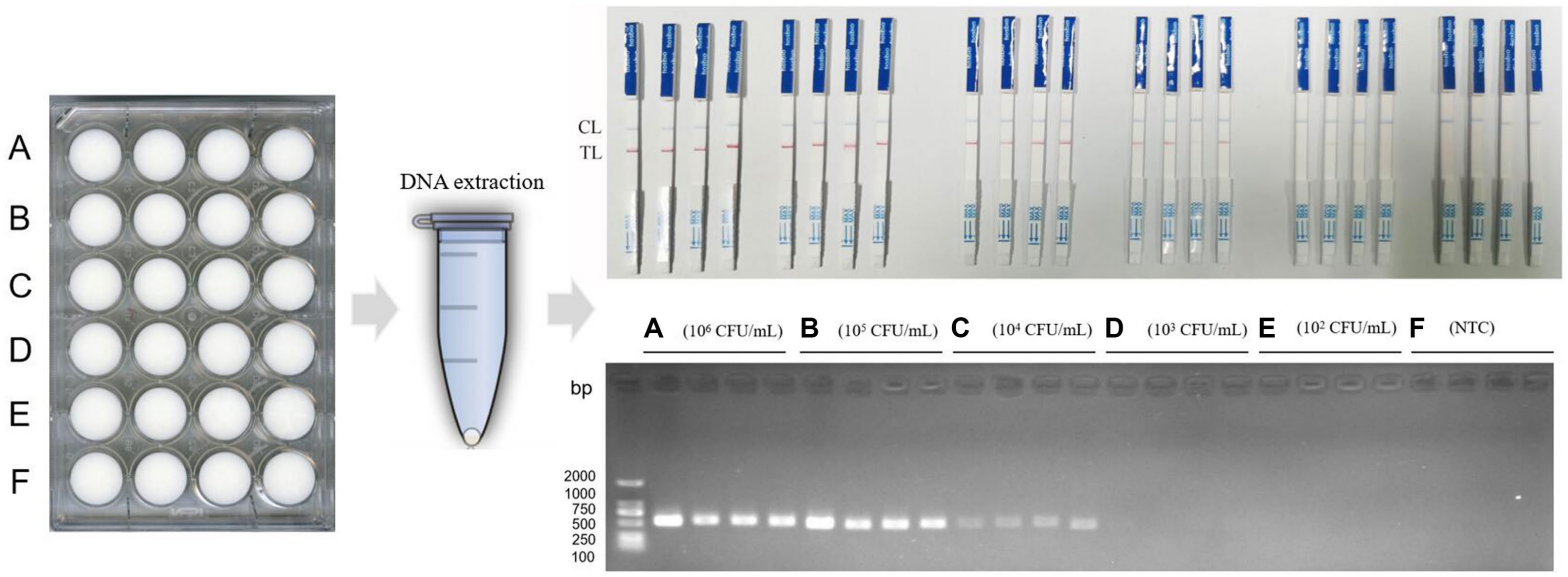
RPA-LFB detection of *C. perfringens* contamination in milk.

In the results of milk samples, when the concentration of artificially contaminated *C. perfringens* was 1 × 10^6^ CFU/mL and 1 × 10^5^ CFU/mL, the results of both methods were positive. When the concentration of contamination was 1 × 10^4^ CFU/mL, the results of RPA-LFB were positive, and the results of PCR electrophoresis bands began to show weak positivity. When the contamination concentration was 1 × 10^3^ CFU/mL, the results of RPA-LFB were positive, and the results of PCR method were negative. Eventually, when the contamination concentration decreased to 1 × 10^2^ CFU/mL, the RPA-LFB was weakly positive for two samples and negative for two, at which point the results were no longer stable (Figure 7). Therefore, RPA-LFB was able to effectively detect *C. perfringens* contamination in milk at a concentration of 1 × 10^3^ CFU/mL.

## 4 Discussion

The majority of *C. perfringens* food poisoning is associated with contaminated food processing and improper storage (28, 29). In the United States, food poisoning outbreaks caused by *C. perfringens* have been reported throughout the year, with the highest numbers in November and December, the months of many holiday parties and events, when people gather to eat foods such as barbecues, juices, and poultry that are often cooked in large batches or prepared in advance. These are also the months with the highest number of outbreaks of *C. perfringens* poisoning caused by beef and poultry (30). In contrast, foodborne disease outbreaks due to most other etiologies were more frequent in summer (31).

Restaurants (43%) were the most common setting for *C. perfringens* food poisoning, followed by private homes (16%) and prisons (11%) (30). Food poisoning caused by *C. perfringens* is often large-scale and causes severe morbidity. Symptoms of *C. perfringens* food poisoning occur 8–18 h after ingestion of contaminated food (32), and the spores of *C. perfringens* are highly thermostable (33), meaning that cooking does not kill it or reduce its pathogenicity. Therefore, the establishment of a rapid method for detecting *C. perfringens* contamination suitable for field testing is essential for food safety and public health.

According to international food safety standards, the detection limit of *C. perfringens* in food is 1 × 10^2^ to 1 × 10^3^ CFU/mL (34). In the absence of an enrichment step, the RPA-LFB established in this study could detect *C. perfringens* contamination in chicken and milk at concentrations of 1 × 10^4^ CFU/mL and 1 × 10^3^ CFU/mL, respectively. Interestingly, the sensitivity of RPA-LFB for milk was higher in this study. The lactose and liquid environment in milk is more favorable for *C. perfringens* proliferation during simulated infections (15). Some studies have reported that the enrichment culture step is beneficial (11), but enrichment culture operations are generally difficult to perform in field testing efforts.

RPA-LFB offers shorter reaction times, simpler handling and more intuitive results than PCR. It can be completed in less than 20 min, with 15 min amplification reaction time and 3 min LFB reading time. The detection time for the PCR method was 110 min, with 90 min for the amplification reaction and 20 min for electrophoresis. However, the RPA-LFB assay for *C. perfringens* in this study still has some limitations in the research process. In this study, we used artificial contamination to simulate the contamination of food by *C. perfringens* to quantitatively control the degree of contamination. In reality, food contamination is often more complex, and may be contaminated by a mixture of pathogenic bacteria at the same time, and is often accompanied by food spoilage and shape change, which makes the detection more difficult and puts forward higher requirements for the extraction method of food nucleic acid. Due to a number of reasons, we are unable to obtain a certain number of food products contaminated with *C. perfringens*, and many food products contaminated under natural conditions have been immediately disposed of and destroyed. In subsequent studies, we will try to conduct validation of food samples contaminated with multiple pathogens and try to collect food products contaminated under natural conditions. More refinements to the RPA-LFB method we have established will be made.

The α-toxin is commonly used as a target for the identification of *C. perfringens*. RPA-LFB targets the *plc* gene, which is highly conserved in the α-toxin (35). Currently, there are a number of methods available for the detection of *C. perfringens*. The real-time PCR Kit Sure-Fast *C. perfringens* Plus (Germany) can detect *C. perfringens* nucleic acid at a minimum concentration of 18 fg/μL. Neumann developed a monoclonal antibody-based method for the detection of *CPE* toxin in *C. perfringens* at a minimum concentration of 1.0 pg./mL (36). Milton developed SRCA method based on saltatory rolling circle amplification for the detection of *C. perfringens* in pork. The method can detect nucleic acid samples at a minimum concentration of 80 fg (37). Dave established a colorimetric method for the detection of *C. perfringens* by detecting the presence of extracellular lecithinase through a PNPC-impregnated probe. The colorimetric method is suitable for on-site detection but requires 1 h to complete the color development assay (38). Many detection methods for *C. perfringens* are superior to the RPA-LFB method in terms of sensitivity. However, the advantage of RPA-LFB is that the detection results are more intuitive, which can be observed directly by naked eyes and the detection time is shorter. Combined with the boiling method or the rapid food genome extraction kit, it is more suitable for on-site detection and has good application prospects.

## 5 Conclusion

In conclusion, the RPA-LFB visual assay using the *C. perfringens plc* gene as the target has the advantages of simple operation and intuitive results, and can be completed within 20 min. It can be used for the rapid detection of *C. perfringens* contamination in chicken meat and milk, and is suitable for on-site detection work.

## Data availability statement

The original contributions presented in the study are included in the article/supplementary material, further inquiries can be directed to the corresponding author.

## Author contributions

RT: Conceptualization, Writing – original draft, Writing – review & editing. FX: Validation, Writing – review & editing, YL: Resources, Writing – review & editing, GL: Resources, Software, Writing – review & editing, QL: Writing – review & editing. JW: Writing – review & editing. HZ: Writing – review & editing. LD: Supervision, Writing – review & editing, WZ: Resources, Supervision, Writing – original draft, Writing – review & editing.

## Footnotes

^1^https://www.premierbiosoft.com/primerdesign/
